# Visual monitoring in real-time improves consistency of 3D IASTM force applied to humans

**DOI:** 10.1186/s12998-026-00668-6

**Published:** 2026-07-23

**Authors:** M. Terry Loghmani, Rachael Powell, George J. Eckert, Sarah Morgan, Abhinaba Bhattacharjee, Stanley Chien, Sohel Anwar

**Affiliations:** 1 School of Health and Human Sciences, Department of Graduate Health Professions, Doctorate of Physical Therapy Program, Indiana University, 1050 Wishard Blvd., IN 46202 Indianapolis, USA; 2 School of Medicine, Department of Biostatistics and Health Data Science, Indiana University, Indianapolis, IN 46202 USA; 3 School of Mechanical Engineering, Purdue University, Indianapolis, IN 46202 USA; 4 School of Electrical & Computer Engineering, Purdue University, Indianapolis, IN 46202 USA

**Keywords:** Instrument-assisted soft tissue manipulation (IASTM), Reliability, Digital technology, Physical therapy, Quantifiable soft tissue manipulation, Manual Therapy

## Abstract

**Background:**

Instrument-assisted soft tissue manipulation (IASTM) is widely used, yet clinicians mostly rely on their subjective perception of applied force which can lead to variability. No studies have determined intra- and inter-examiner reliability of three-dimensional (3D) IASTM force–motions applied to humans. The objective of this study was to evaluate whether real-time visual monitoring enhances consistency of applied IASTM force.

**Methods:**

This study was an observational reliability study with blinded assessors. 45 healthy adults were enrolled between June to December 2021. Clinicians (two novice, two experienced) applied 1-inch IASTM linear strokes using two quantifiable soft tissue manipulation (QSTM) smart devices (localized; dispersive) to lumbar and calf regions under two conditions: (1) applying self-perceived “medium” force without visual monitoring, and (2) applying force guided by real-time visual monitoring from a graphic visual interface (GVI). Triaxial (3D) average peak force (primary variable), stroke frequency, and angle were measured. Linear mixed models and variance components evaluated repeatability and reproducibility within and between clinicians and across two sessions.

**Results:**

Visual monitoring substantially reduced variability in average peak force across clinicians, regions, devices, and sessions. Standard deviations (SDs) were significantly larger and ranges meaningfully broader without monitoring compared to with monitoring. Monitoring improved intra- and inter-examiner consistency by ≥ 30% in nearly all conditions, with medium to very large effect sizes. Effects on stroke frequency were mixed, and influence on angle minimal.

**Conclusions:**

Visual monitoring in real-time significantly improved the consistency of 3D IASTM force applications on humans. Optimal reliability is foundational to practice fidelity, training, and more rigorous investigation of dose–force response relationships in manual therapy.

*Trial Registration*: The study was prospectively registered at ClinicalTrials.gov (Identifier: NCT04923633).

## Background

Musculoskeletal (MSK) disorders are pervasive in the United States affecting millions of individuals, more than any other chronic condition [1, 2]. People look for non-invasive and non-pharmacological approaches to alleviate their pain and functional limitations [3]. Manual therapy is a force-based manipulation (FBM), a conservative approach frequently used by clinicians to apply mechanical forces to the intact surface of the body for therapeutic intent through the processes of mechanotransduction [4]. Despite long-time and widespread use, understanding of FBM mechanisms and optimization of its clinical outcomes are not fully realized [5–8].

Soft tissue manipulation (STM) is a common type of FBM; a massage-based modality that can be delivered by hand alone or instrument-assisted (IASTM) [9–12]. IASTM is a form of mechanotherapy demonstrating a spectrum of benefits, including attenuating pain, mitigating inflammation, and promoting functional change [13–16]. However, IASTM relies mostly on subjective parameters, lacking a clinically feasible means to objectively characterize applied forces during various motion techniques, hindering its reliability, reproducibility, and systematic comparison, thereby impeding evidence-based practice [17–19].

The National Institutes of Health (NIH) recognizes the need to better characterize FBM approaches and to quantify manual therapy force-motions to promote mechanisms research and optimize clinical outcomes [20]. Varying force levels produce distinct biological effects, underscoring the clinical importance of reliable manual therapy force-motion delivery [21]. Dynamic force-time parameters in spinal manipulation have been characterized to some degree to better enable dose-response studies [22, 23]. A multitude of factors can influence IASTM force-motion application including patient condition, anatomical region, and technique approach [24, 25]. Accordingly, measurable parameters should account for the complexity of IASTM force-motion applications while complementing clinician perception and client perspective within a collaborative care model. IASTM reliability is a prerequisite for linking dynamic force and motion parameters to patient outcomes.

In related research, robotic massage-like devices and other approaches, such as affixed flexible sensors, have been used to quantify IASTM forces, including animal models and instrumented human applications [18, 26–27]. IASTM reliability research comparing hand-grips and device type has largely relied on skin simulants mounted to force plates to record uniaxial compressive forces under standardized conditions [28–33]. These methods provide initial insight into force magnitude; however, gaps remain, as they may not account for multidirectional loading, motion irregularities, or the practical demands of patient care. Thus, little is known about within or between clinician reliability of 3-dimensional (3D) IASTM force as applied to humans.

**Fig. 1:**
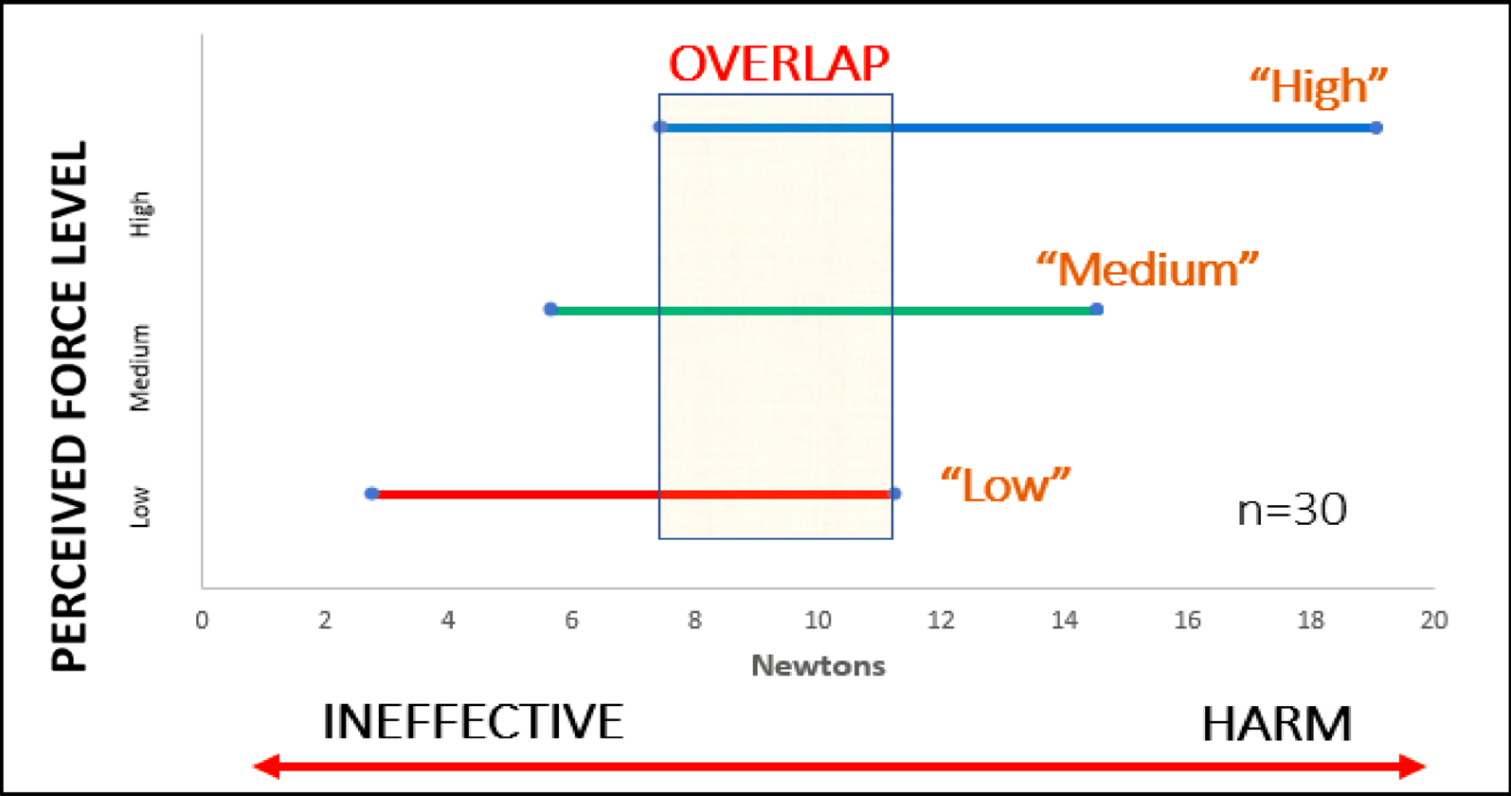
Overlap in Levels of Self-Perceived Force Applications on an Inanimate Pad. Conceptually, too low of a force could be ineffective while too high may be harmful

In preliminary work, 30 doctor of physical therapy (DPT) students were trained using a quantifiable soft tissue manipulation medical device system (QSTM^®^, Precision Care Technologies, Inc., Indianapolis, IN) [34, 35] to apply self-perceived localized force levels of “high,” “medium,” and “low” in random order using 1-inch linear strokes (averaged 5 trials/force level) at a rate of 1 Hz, against a smooth, inanimate padded surface [36]. The 3D average peak force applied was captured by the device system. Notable overlap was found, with some participants’ self-perceived “low” corresponded to others’ perception of “high” force magnitude application (Fig. 1). This conflict in individuals’ force perception is important since, although not proven for these specific force levels, too low a force may be ineffective, while too high could lead to harm.

**Fig. 2:**
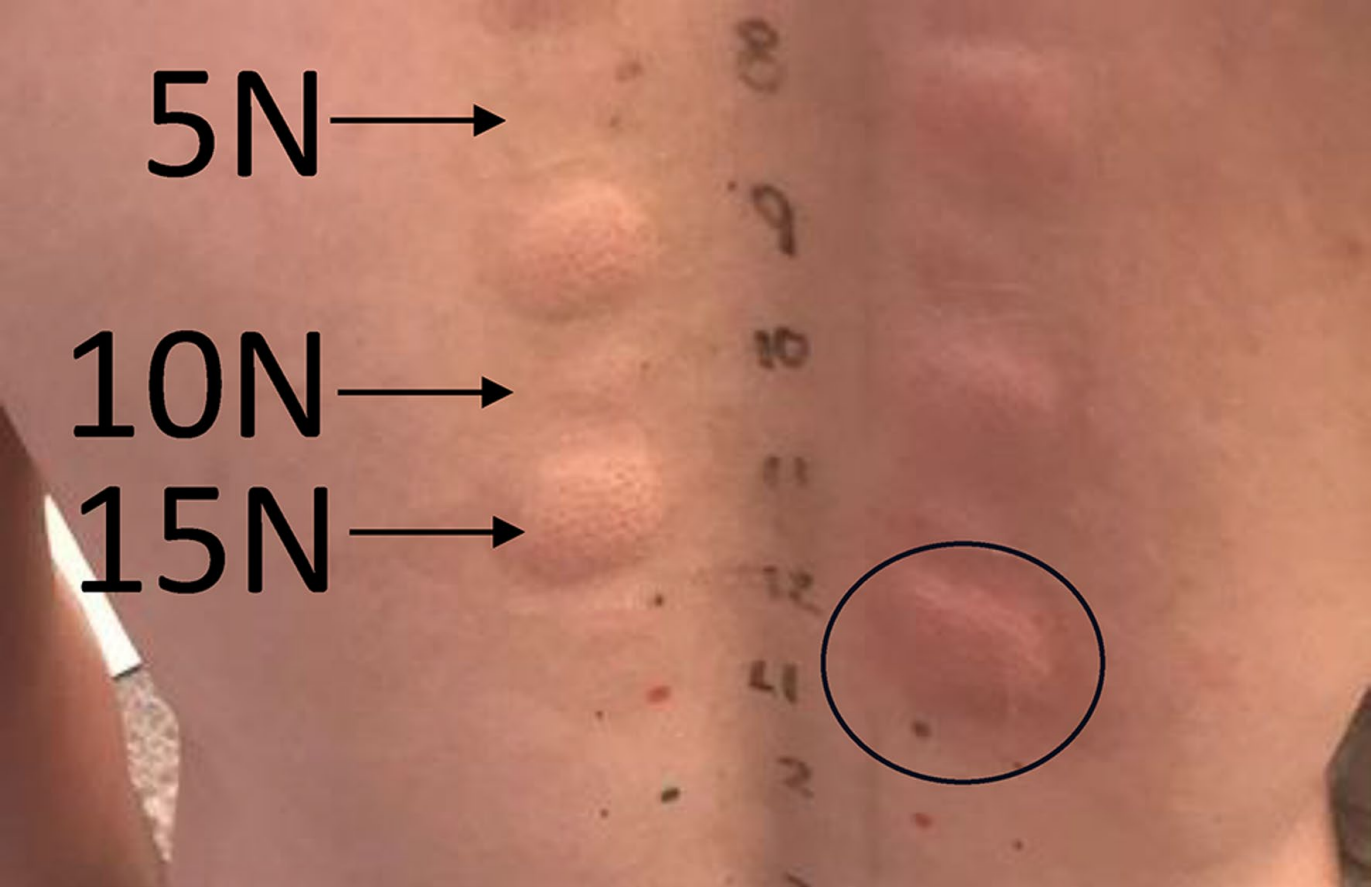
Superficial Tissue Response to Different IASTM Force Magnitude Levels. The superficial tissue in the lower thoracolumbar region on this 21 y.o. male responded with greater welting and redness at progressively higher levels of force (arrows) applied in random order for 15 s/linear stroke. The most marked response (circle) corresponded with his self-reported site of tenderness

In other preliminary work, distinct differences in superficial tissue response were observed when 1-inch linear strokes were applied for 15 s in random order by one clinician to marked regions on the back of the same healthy human male model (21 years old) while visually monitoring force as applied on the QSTM GVI. This allowed force to be standardized at various levels (low 5 N; medium 10 N; high 15 N) based on prior testing of the model’s force self-perception. The subject’s immediate visible tissue response depicted in Fig. 2 points to potential implications of differing IASTM force applications on biological effects and patient experience.

Subsequent testing between two licensed practicing physical therapists (each with > 8yrs IASTM experience) demonstrated a 25% difference (5 *N* ± 1.4 N) in average peak force when asked to apply self-perceived “high” force (*n* = 30 trials) (15 s/trial) to an inanimate pad using 1-inch IASTM linear strokes. When they were allowed to visually monitor the targeted force (15 N, as previously tested), only a 3.03% difference (0.5 *N* ± 1 N) was found, suggesting visual monitoring may reduce variability.

This study aims to address a major gap in the evidence for IASTM manual therapy practice. The purpose was to explore whether real-time visual monitoring enhances the consistency of IASTM force applied to humans. To test the hypothesis that monitoring improves the consistency of IASTM 3D force application, reliability was evaluated within (intra-examiner repeatability) and between (inter-examiner reproducibility) novice and experienced clinicians, both within and across sessions (test–retest reliability). Testing included two devices in different body regions under two conditions: with and without visual force monitoring. The overarching goal is to inform soft tissue manual therapy practice.

## Methods

An observational, repeated-measures reliability study was conducted at a single site (Indiana University, Indianapolis, IN, USA) using a convenience sample of healthy participants. The protocol procedures and human subject protection plan were approved a priori by the Indiana University Institutional Review Board (IRB approval # 10329). The study was registered under ClinicalTrials.gov (Identifier: NCT04923633). It was supported by the NIH National Center for Complementary and Integrative Health (NCCIH) (FAIN# R41AT011494). Contents and conclusions expressed in this publication are solely the responsibility of the authors and do not necessarily represent the official views of the NIH or NCCIH.

### Subjects

Forty-five healthy adult participants (18–35 years old) with a body mass index (BMI) between 18.5 and 30 kg/m^2^ were enrolled between June to December 2021 according to established exclusion and inclusion criteria and informed consent obtained. Children and individuals classified as underweight or obese were excluded to reduce variability attributable to subject characteristics.

### Instrumentation

QSTM was used to capture and record 3D IASTM force-motion data in real-time as manually applied by clinicians to human participants. This manual therapy technology is described in detail in the literature, validating its design, force-motion measurement reliability against external scales, usability on humans [37–40], and on animals for standardization of force [13]. The system comprises handheld, force-sensing device applicators (Q1-L localizing; Q2-D dispersive) with embedded tri-axial load cells to measure 3D IASTM force. Motion is captured using an inertial measurement unit (IMU), including an accelerometer for tri-axial linear acceleration and a gyroscope for angular orientation. Device firmware performs sensor acquisition, signal filtering and processing, calibration, and real-time communication with custom software (Q-Ware©, copyright of Indiana University Board of Trustees) on a personal computer. Data is displayed on a GVI to aid visual monitoring of force-motion data. Each device is placed in its respective calibration cradles at the start of a treatment session and when switching between use. Captured data are stored in a record system and exported to Microsoft Excel for processing.

### Procedures

Four female clinicians, including two DPT students (novices) and two experienced physical therapists, were trained using the QSTM device system. A novice was defined as a clinician with ≤ 1 year and an experienced with ≥ 8 years of clinical experience [41]. All were previously trained in IASTM (Graston Technique, Indianapolis, IN). All female clinicians, neither obese nor underweight, were used to reduce potential variability introduced by clinician attributes. All testing was conducted in the same heat-controlled room.

**Fig. 3:**
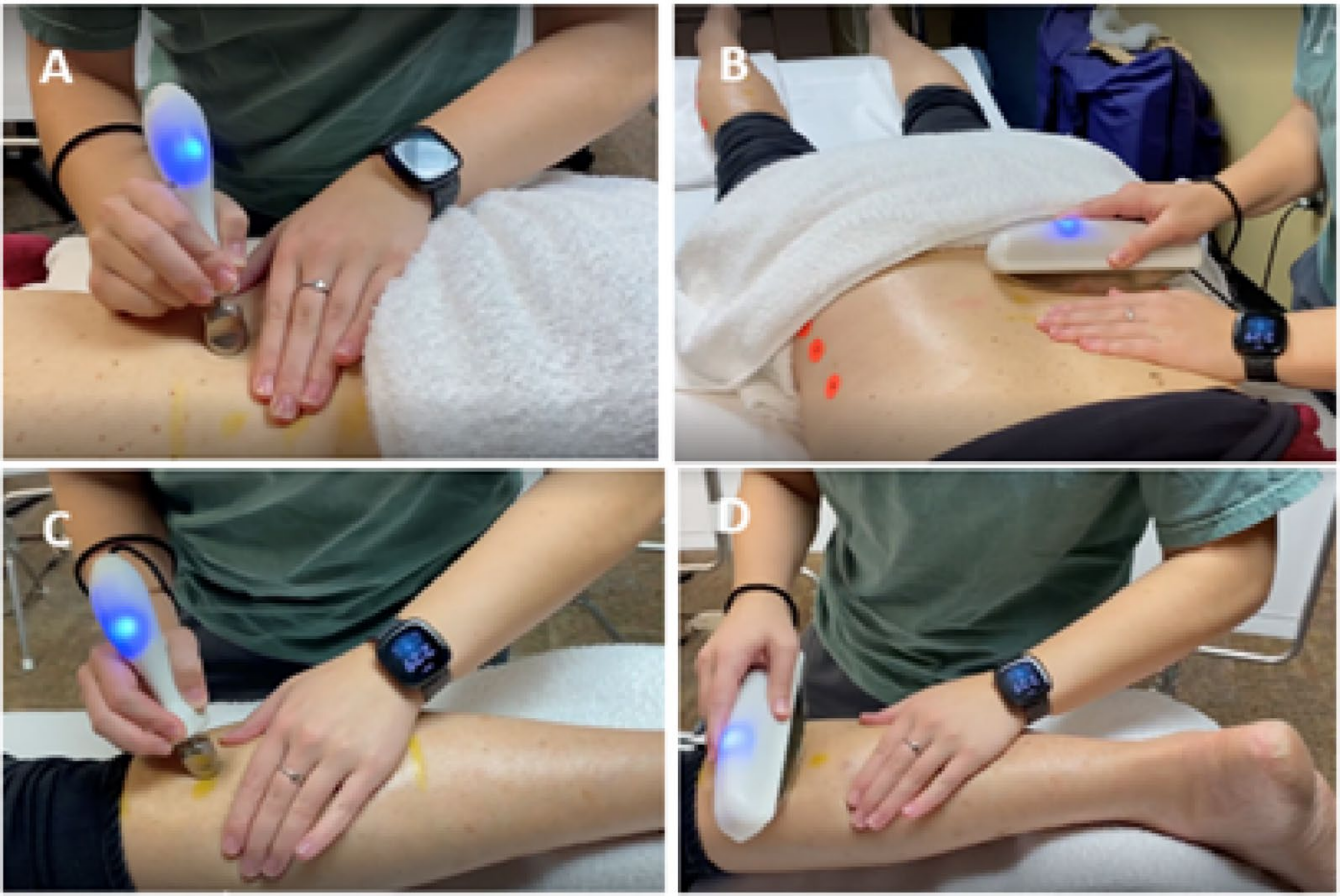
Quantitative IASTM Applications. Q1-L for localized (**A** &** C**) and Q2-D for dispersive (**B** &** D**) force applications to the low back and calf areas

Subjects were instructed to lie prone on a power-adjustable treatment table in a standardized position, with a pillow under their torso and a bolster under their ankles for comfort. The table height was adjusted to a standardized height, level to each clinician’s greater trochanter. For each subject, an online number generator was used to randomly assign the clinician, device, and trial site sequences, reducing bias related to order.

Clinicians applied 1-inch IASTM linear strokes using QSTM to standard sites marked lateral to the L1, L3, and L5 spinous processes in the lumbar region, and to the superior, middle, and inferior thirds of the gastrocnemius in the calf region. The Q1-L applicator (100 N range; 100 Hz monitoring frequency) was used to manually apply and sense *localized* forces perpendicular to the fiber alignment in small areas (2.5 × 2.5 cm) in each region (Fig. 3A and C). The Q2-D device (200 N range; 100 Hz monitoring frequency) with a broad blade was used to apply *dispersive* forces parallel to the fiber alignment over larger areas (2.5 × 7.6 cm) in both body regions (Fig. 3B and D). An emollient was used to reduce friction. Three stroke application trials were averaged (10 sec/trial; 30-second rest between trials; 10 min rest between clinicians) per region and device. This is considered a non-therapeutic treatment dose (i.e., < 1 min total/site per Graston Technique). All strokes were applied within subject tolerance. Clinicians were allowed to use their preferred stroke frequency, grip, and angle in alignment with their naturalistic style.

**Fig. 4:**
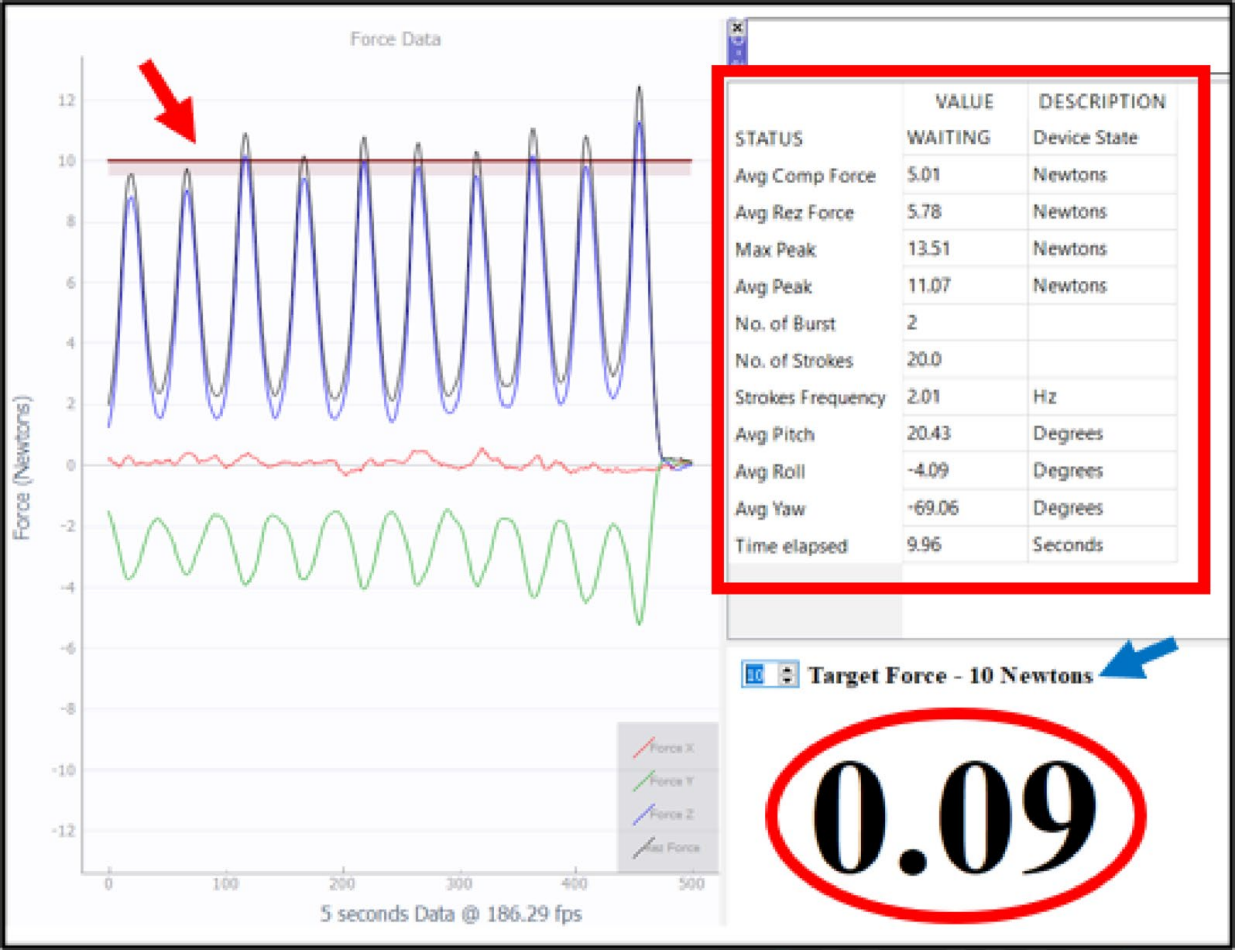
Representative GVI Display of Real-Time IASTM 3D Force-Motion Tracking and Data Acquisition. On the Force Data Graph, the average magnitude of the root mean square (RMS) force vector of the 3D force (N) components (absolute values) forms the resultant force (black line) of every force-motion stroke cycle applied during treatment. The load cell measurements are transformed into 3 force components: compressive (Vertical-Z, blue line) normal to the treatment plane, and planar directions from the point of contact (Lateral-X, red line; Longitudinal-Y, green line). Negative angle values indicate directionality. A pre-determined Target Force (blue arrow) can be set to provide visual feedback on the graph at the target line (red arrow). Instantaneous force readouts can also be monitored (red circle). A Treatment Report (red box) for force-motion data is generated once the device is clicked off treatment mode into an idle state

Clinicians applied their self-perceived “medium” level of force without monitoring the GVI feedback, followed by a 30-min break before the next testing condition when they applied a targeted force while periodically (self-paced) monitoring the GVI. Clinicians were instructed to apply the targeted force levels as follows: “Please apply the targeted force while monitoring the visual display at your discretion. Continuous monitoring is not required.” This testing order was used to avoid potential bias from visual monitoring first. Clinicians were blinded to their own and each other’s performance records and trial averages for the duration of the study. A representative illustration of the GVI clinicians used to monitor targeted force application is depicted in Fig. 4.

The targeted medium force magnitude was set at 10 N for Q1-L and 20 N for Q2-D. Targeted force magnitudes were based on preliminary testing of the clinicians’ average perceived “medium” force for each device. The 3D average peak force, i.e., “treatment force” (N) was the primary variable of interest, while stroke frequency (“rate”; Hz), and inclination angle (“treatment angle”; °) were secondary parameters captured by the system and later analyzed.

One novice and one experienced clinician repeated this process 5–7 days later to assess test-retest repeatability. All subjects (*n* = 45) returned to Session 2 and the same procedures were followed. After both visits, subjects were given an ice pack and instructed on standard stretches to minimize potential soreness.

### Statistical analysis

Data recording, collection and entry were conducted independently by research assistants and data analysis was performed by an independent biostatistician to reduce potential or perceived bias. Sample size was estimated based on prior testing and the literature to achieve ≥ 80% power to detect a monitoring effect [42, 43]. Descriptive statistics (i.e., mean, (SD), range [high, low]) for primary (average peak force) and secondary (stroke frequency, angle) parameters under each monitoring condition for both devices (Q1-L, Q2-D) and body regions (back, calf) for all clinicians (A, B, C, D) for Session 1 and for two clinicians (one novice, B; one experienced, C) from Session 2 were calculated. Comparisons of measurements with monitoring versus without monitoring of the applied force and between the Q1-L and Q2-D devices were made using linear mixed models that included random effects to account for within-subject and within-examiner correlations among measurements. Means and SDs for each parameter were used to estimate the following variance components: between sessions, examiners, body locations, and trials within a session at each region. Confidence intervals (95% CI) were calculated to compare monitoring vs. without monitoring conditions and Q1-L vs. Q2-D devices. Cohen’s D and Glass’s Δ were calculated to determine effect sizes. Cohen’s D is the convention for effect size, though it assumes similar variability and may not best capture differences in consistency, while Glass’s Δ uses monitoring as the reference to express deviations relative to its expected outcome of greater consistency under this condition. Intra-examiner repeatability and inter-examiner reproducibility were evaluated by analyzing the variance components of the measurements, estimating variability between sessions, examiners, and trials within a session. Variance components were evaluated separately for both devices with and without visual monitoring of the targeted force. Intraclass correlation coefficients (ICCs) were not appropriate to evaluate variability in this study design because the ICC calculations rely on variability across study subjects, while in this study the design planned for relatively constant force applied across subjects. A statistical package was used (SAS v9.4). Bland-Altman plots were used to visualize repeatability and agreement. Significance levels were set at *p* < 0.05.

## Results

Forty-five subjects (36 females, 9 males) participated. The mean age, SD, and range [high, low] was 23.90 (2.33) [19.0, 35.0] years old and BMI 23.84 (2.61) [19.5, 29.0]. Data was assessed for normality and any outliers attributable to equipment measurement or recording error were excluded from the analysis.

### Description of IASTM parameters

Descriptive statistics for IASTM force-motion parameters as captured using both devices in each region with (W) and without (WO) monitoring are summarized in Table 1. Overall, greater precision in average peak force applications was achieved with monitoring as evidenced by smaller SDs and narrower spread in ranges compared to self-perceived applications without monitoring, while the impact on stroke frequency and angle was mixed and less pronounced.

**Table 1:** IASTM Force-Motion Parameters Summary. Descriptive statistics for force-motion parameters (3D average peak force, N; stroke frequency, Hz; and inclination angle, °, during Session 1 for all clinicians are summarized (mean; (SD); range [high, low]), both W and WO visual monitoring, for each device (Q1-L; Q2-D), in each body region (Back, Calf). Absolute differences (Abs Difference) [W, WO] in the means, SDs, and ranges are included along with percent (%) change [W to WO] to support interpretation

Parameters	Location	Device	W Monitoring	WO Monitoring	Abs Difference [W, WO]	% Change [W to WO]
Average Peak Force (N)	Back	Q1-L	9.81 (0.35) [10.6, 8.8]	11.12 (1.52) [15.5, 7.6]	1.31 (1.17) [1.8, 7.8]	13.4 (334.3) [341.8]
	Back	Q2-D	19.87 (0.88) [22.8, 9.1]	23.06 (3.91) [46.5, 9.6]	3.20 (3.03) [13.7, 36.9]	16.1 (344.4) [169.0]
	Calf	Q1-L	9.64 (0.25) [10.5, 8.3]	9.66 (1.38) [14.6, 4.6]	0.02 (1.13) [2.2, 10.0]	0.2 (452.0) [353.9]
	Calf	Q2-D	19.63 (0.56) [21.3, 16.9]	20.99 (3.56) [39.9, 9.0]	1.37 (3.00) [4.4, 31.0]	6.9 (535.7) [602.3]
Stroke Frequency (Hz)	Back	Q1-L	2.13 (0.18) [2.9, 1.0]	1.81 (0.16) [2.4, 1.2]	0.32 (0.02) [1.9, 1.2]	-15.0 (-11.1) [-40.0]
	Back	Q2-D	2.54 (0.25) [3.1, 1.4]	2.06 (0.26) [3.2, 1.1]	0.48 (0.01) [1.6, 2.1]	-18.9 (4.0) [25.6]
	Calf	Q1-L	2.12 (0.15) [2.7, 1.6]	1.83 (0.19) [2.9, 1.1]	0.28 (0.04) [1.1, 1.8]	-13.7 (26.7) [59.3]
	Calf	Q2-D	2.48 (0.21) [3.1, 1.9]	2.19 (0.24) [3.1, 1.3]	0.30 (0.03) [1.2, 1.8]	-11.7 (14.3) [46.8]
Angle (°)	Back	Q1-L	45.94 (8.39) [121.3, 23.6]	44.56 (7.19) [72.9, 10.0]	1.38 (1.20) [97.7, 62.9]	-3.1 (-14.3) [-35.6]
	Back	Q2-D	103.50 (27.60) [140.0, 16.8]	100.10 (27.80) [141.2, 15.0]	3.41 (0.17) [123.9, 126.3]	-3.3 (0.7) [1.9]
	Calf	Q1-L	60.53 (10.19) [110.1, 32.1]	59.43 (10.31) [103.8, 26.6]	1.10 (0.12) [78.1, 77.2]	-1.8 (1.2) [-1.2]
	Calf	Q2-D	88.61 (33.72) [147.2, 15.3]	93.76 (29.97) [138.7, 15.4]	5.15 (3.75) [132.0, 123.3]	5.8 (-11.1) [-6.6]

#### Force

The average peak force mean absolute difference (% change) was between 0.02 N (0.1%) to 3.20 N (16.1%) higher without monitoring, depending on the region and device. SD values were consistently larger (1.13 N to 3.03 N absolute difference) while range differences demonstrated a wider spread without monitoring, reflecting greater variability of applied forces.

#### Rate

Stroke frequency (rate) was 0.28 Hz (13.7%) to 0.48 Hz (18.9%) slower without monitoring under all conditions. Similar to force, without monitoring the SDs and ranges for stroke frequencies were higher, except for Q1-L to the back.

#### Angle

Smaller effects on the angle of applications were found between monitoring conditions. SDs varied and wide ranges occurred under all conditions (62.9° to 132.0° absolute difference).

### Comparison of IASTM parameters without versus with monitoring

Results of mean differences in force-motion parameters with 95% CI for monitoring comparisons are summarized in Table 2. Clinicians applied significantly higher average peak force (N) without monitoring than with monitoring for both devices and body regions (*p* < 0.001) with medium to very large effect sizes indicating substantial practical significance, except for Q1-L to the calf. Clinicians applied a significantly slower stroke frequency without monitoring than with monitoring for each device in both body regions (*p* < 0.001), with very large effect sizes. Monitoring had insignificant, negligible effects on angle.

**Table 2:** Monitoring Comparisons: Mean Differences in Force-Motion Parameters (95% CI). Values are presented as mean (95% CI) for each condition, with differences calculated as WO monitoring minus W monitoring. Effect sizes are expressed as Cohen’s d and Glass’s Δ

Parameters	Location	Device	WO Monitoring Mean (95% CI)	W Monitoring Mean (95% CI)	Difference (95% CI)	*p*-value	Effect Size (Cohen’s D; Glass’s Δ)
Average Peak Force (N)	Back	Q1-L	11.12 (9.63 to 12.57)	9.81 (8.34 to 11.29)	1.29 (0.97 to 1.60)	< .001	1.13; 3.76
	Back	Q2-D	23.06 (21.59 to 24.53)	19.87 (18.39 to 21.34)	3.20 (2.88 to 3.51)	< .001	0.60; 2.80
	Calf	Q1-L	9.66 (8.18 to 11.13)	9.64 (8.17 to 11.12)	0.01(− 0.30 to 0.33)	0.940	0.01; 0.02
	Calf	Q2-D	20.99 (19.51 to 22.46)	19.63 (18.16 to 21.10)	1.36 (1.04 to 1.68)	< .001	0.73; 2.53
Stroke Frequency (Hz)	Back	Q1-L	1.81 (1.65 to 1.97)	2.12 (1.97 to 2.28)	− 0.32 (− 0.35 to − 0.28)	< .001	− 1.18; − 1.25
	Back	Q2-D	2.05 (1.90 to 2.21)	2.53 (2.38 to 2.69)	− 0.48 (− 0.52 to − 0.45)	< .001	− 1.19; − 1.63
	Calf	Q1-L	1.83 (1.68 to 1.99)	2.12 (1.96 to 2.27)	− 0.28 (− 0.32 to − 0.25)	< .001	− 2.89; − 2.07
	Calf	Q2-D	2.19 (2.03 to 2.34)	2.48 (2.32 to 2.64)	− 0.29 (− 0.33 to − 0.26)	< .001	− 1.59; − 2.13
Angle (°)	Back	Q1-L	44.27 (38.06 to 50.48)	45.94 (39.75 to 52.14)	− 1.67 (− 6.31 to 2.97)	0.479	− 0.12; − 0.12
	Back	Q2-D	99.82 (93.62 to 106.03)	103.53 (97.32 to 109.73)	− 3.70 (− 8.35 to 0.95)	0.118	− 0.13; − 0.14
	Calf	Q1-L	58.91 (52.69 to 65.12)	60.56 (54.35 to 66.77)	− 1.65 (− 6.32 to 3.01)	0.487	− 0.09; − 0.09
	Calf	Q2-D	93.48 (87.27 to 99.69)	88.61 (82.41 to 94.81)	4.87 (0.23 to 9.51)	0.040	0.13; 0.15

### Device comparison

As expected, Q1-L (localized) applications were significantly less than Q2-D (dispersive) for average peak force, stroke frequency, and angle, with or without monitoring. All p-values were *p* < 0.001.

### Regional comparison

Average peak force was significantly higher for the back than calf for both Q1-L and Q2-D (*p* < 0.05). Stroke frequency did not differ by device between the back and calf for Q1-L with or without monitoring (*p* > 0.40) or for Q2-D with monitoring (*p* = 0.12) but was slower in the back than calf without monitoring (*p* < 0.001). Angle was significantly lower in the back than calf for Q1-L but, in contrast, lower for the calf than back for Q2-D (*p* < 0.001).

### Examiner type comparison based on levels of experience

No significant differences were found in IASTM force-motion applications based on experience level.

### Evaluation of repeatability/reproducibility

**Fig. 5:**
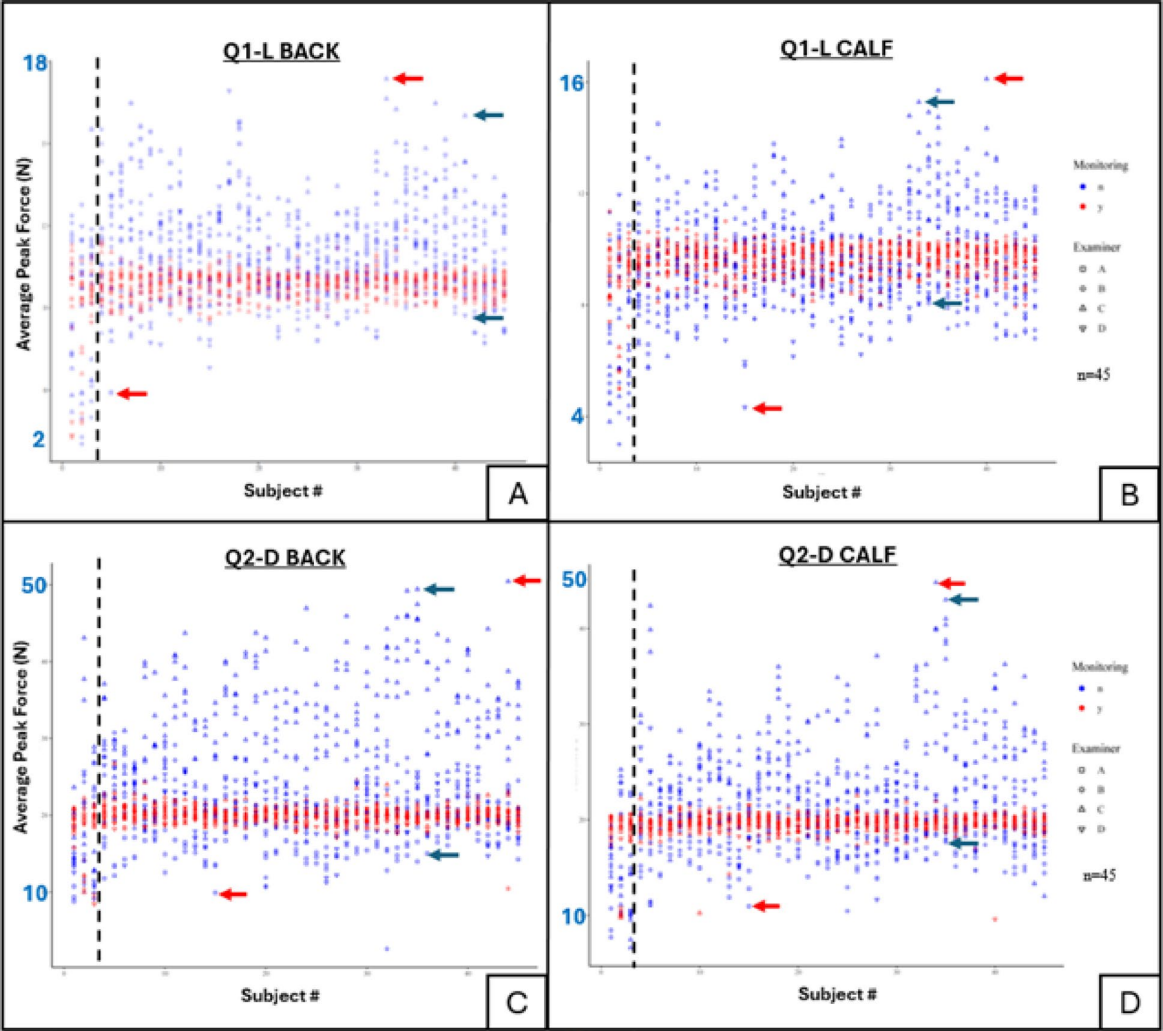
Consistency & Reproducibility of IASTM Force Application With and Without Monitoring. Representative Bland-Altman plots illustrate improved reliability of Average Peak Force application with visual monitoring (tight band of red points) compared to wide variance without monitoring (blue points) during localized (Q1-L) IASTM force applied to the back (**A**) and calf (**B**) and dispersive (Q2-D) to the same regions (**C** &** D**) for all clinicians (different shaped point for each). Beyond a potential learning curve (before dotted line), the red arrows depict a spread between maximum and minimum values that occurred without visual monitoring across subjects, while the blue arrows depict representative spreads of maximum and minimum values without monitoring between clinicians within the same subject. For example, in (**C**) without monitoring there was 40.0 N difference across subjects (red arrows), while a 35.0 N spread occurred within a given subject (blue arrows) compared to less than 5 N variance across and within subjects with visual monitoring

Analysis of the variance components measurements revealed monitoring significantly reduced variability in IASTM average peak force applications by a clinically meaningful improvement of ≥ 30.0% [44, 45] under all conditions. Variability was also reduced for stroke frequency (summarized in Table 3). Interestingly, between examiner reliability for angle of application mostly worsened with monitoring. Representative Bland-Altman plots visually depict significantly improved precision and consistency between all examiners with both devices in each region (Fig. 5).

**Table 3:** IASTM Repeatability/Reproducibility - Percent Change in Variance Components. Within-session, between-session, and between-examiner variability are expressed as SDs for conditions W and WO monitoring across regions and devices (Q1-L, Q2-D).

		Q1-L				Q2-D			
		Average Peak Force (N)		Stroke Frequency (Hz)		Average Peak Force (N)		Stroke Frequency (Hz)	
		Back	Calf	Back	Calf	Back	Calf	Back	Calf
*Within-Session SD*									
Examiner A	W Monitoring	0.27	0.21	0.09	0.07	0.46	0.45	0.16	0.15
	WO Monitoring	0.85	0.71	0.08	0.07	1.60	1.30	0.20	0.16
	**% Change**	**− 68.2%**	**− 70.4%**	**9.0%**	**− 5.0%**	**− 72.0%**	**− 65.4%**	**− 18.0%**	**− 6.3%**
Examiner B, Session 1	W Monitoring	0.25	0.29	0.08	0.08	0.96	0.65	0.14	0.11
	WO Monitoring	0.76	0.63	0.08	0.07	2.96	2.01	0.20	0.14
	**% Change**	**− 67.1%**	**− 54.0%**	**7.0%**	**11.0%**	**− 67.6%**	**− 67.7%**	**− 30.0%**	**− 23.0%**
Examiner B, Session 2	W Monitoring	0.17	0.21	0.09	0.09	0.54	0.53	0.14	0.15
	WO Monitoring	0.65	0.67	0.08	0.08	1.72	2.11	0.18	0.15
	**% Change**	**− 73.8%**	**− 68.7%**	**10.0%**	**13.0%**	**− 68.6%**	**− 74.9%**	**− 23.0%**	**− 2.0%**
Examiner C, Session 1	W Monitoring	0.51	0.41	0.17	0.15	1.06	1.13	0.28	0.23
	WO Monitoring	1.23	1.12	0.16	0.17	3.54	3.06	0.14	0.16
	**% Change**	**− 58.5%**	**− 63.1%**	**6.3%**	**− 7.0%**	**− 70.1%**	**− 63.1%**	**93.0%**	**45.0%**
Examiner C, Session 2	W Monitoring	0.45	0.62	0.18	0.14	1.18	0.81	0.27	0.22
	WO Monitoring	1.15	1.20	0.15	0.21	3.63	2.70	0.15	0.16
	**% Change**	**− 60.9%**	**− 48.3%**	**15.0%**	**− 34.0%**	**− 67.5%**	**− 70.0%**	**76.0%**	**44.0%**
Examiner D	W Monitoring	0.31	0.49	0.13	0.11	0.60	1.10	0.18	0.16
	WO Monitoring	1.05	0.84	0.16	0.15	2.29	1.95	0.26	0.20
	**% Change**	**− 70.5%**	**− 41.7%**	**− 14.0%**	**− 31.0%**	**− 73.8%**	**− 43.6%**	**− 29.0%**	**− 18.0%**
*Between-Session SD*									
Examiner B	W Monitoring	0.57	0.56	0.17	0.17	1.25	1.17	0.20	0.19
	WO Monitoring	1.61	1.23	0.12	0.12	3.45	2.90	0.26	0.22
	**% Change**	**− 65.2%**	**− 54.3%**	**39.0%**	**50.0%**	**− 63.8%**	**− 59.7%**	**− 24.0%**	**− 13.6%**
Examiner C	W Monitoring	0.57	0.66	0.22	0.19	1.48	1.42	0.33	0.25
	WO Monitoring	1.64	1.62	0.20	0.25	6.28	4.74	0.21	0.21
	**% Change**	**− 66.0%**	**− 60.0%**	**10.0%**	**− 23.0%**	**− 76.4%**	**− 70.0%**	**57.1%**	**21.0%**
*Between-Examiner SD*									
	W Monitoring	0.55	0.48	0.25	0.27	1.14	0.64	0.30	0.25
	WO Monitoring	1.55	1.60	0.31	0.40	8.10	5.13	0.54	0.41
	**% Change**	**− 64.5%**	**− 70.0%**	**− 18.0%**	**− 32.5%**	**− 85.9%**	**− 87.5%**	**− 45.0%**	**− 40.0%**

Percent (%) change (bold) reflects the relative difference in SD between monitoring conditions

#### Within-examiner consistency

Intra-examiner repeatability for average peak force application was higher with monitoring, consistent with lower variability. During Session 1, variability in average peak force within examiners was reduced with monitoring when using Q1-L in the back / calf by 58.5–70.5% / 41.7–70.4%, and for Q2-D by 67.6–73.8% / 43.8–67.6%. Variability in stroke rate decreased for some but increased for other examiners (Q1-L -14.0 to 9.0% / -31.0 to 11.0%; Q2-D -30.0 to 93.0% / -23.0 to 45.0%). During Session 2, clinicians B (novice) and C (experienced) had similar within-examiner test-retest consistency patterns to Session 1 for both force and rate.

#### Between-session test-retest repeatability

Monitoring was associated with lower variability in force measurements for both examiners (B & C) during repeat testing across Sessions 1 and 2. With monitoring, variability in average peak force was reduced using Q1-L in the back / calf by 65.2–66.0% / 54.3–60.0% and with Q2-D by 63.8–76.4% / 59.7–70.0%.

#### Between-examiner reproducibility

Inter-examiner variability for average peak force and stroke rate lowered with monitoring. Variability in average peak force between examiners was reduced when using the Q1-L device in the back / calf by 64.5% / 70.0% and for Q2-D by 85.9% / 87.5%. Reduced variability in stroke frequency between examiners was found to a lesser degree with monitoring for Q1-L by 18.0% / 32.5% and with Q2-D by 45.0% / 40.0%. Greater variability in angle was found with monitoring for Q1-L by 20% / 6% but mixed with Q2-D by -24% / 30%.

## Discussion

This observational reliability study examined how visual monitoring influences intra-examiner repeatability (consistency), inter-examiner reproducibility (agreement), and test-retest reliability of IASTM force-motion parameters applied to healthy human subjects by novice and experienced clinicians for different devices (Q1-L, Q2-D), body regions (back, calf), and Sessions (1 and 2). Using a GVI to monitor force in real-time significantly improved the reliability of IASTM compared to self-perceived “medium” force applications, both within and between examiners, and across sessions regardless of experience level. This significant improvement occurred for both devices, body regions, and sessions. In contrast, monitoring targeted force had less influence on the consistency of stroke frequency or angle.

This is the first known work examining both intra- and inter-examiner reliability of 3D IASTM force–motion parameters as applied to humans. Prior research has relied mostly on intra-examiner reliability using force plates to capture uniaxial compressive forces during simulated treatments [2, 8, 46]. By incorporating 3D force measurement in human participants, the present study may help to advance methodological rigor and relevance in the clinical environment.

Notably, clinicians applied substantially higher average peak force without monitoring than with monitoring, except for Q1-L on the calf, at significantly slower stroke frequencies. Effect sizes ranged from moderate to very large, underscoring strong practical significance. Improving reliability in IASTM applications may have meaningful implications for patient outcomes by reducing unnecessary variability, better correlating dose with outcomes, and enabling more precise tracking of dose–force response over the course of care.

Large effect sizes were observed for mean differences in force–motion parameters (95% CI), primarily because visual monitoring produced more consistent force and stroke frequency with minimal variability. Monitoring narrowed SDs and reduced range dispersion, promoting stability in force application. This reflects a clinically relevant improvement in consistency and repeatability. In contrast, the absence of monitoring consistently resulted in higher force magnitudes compared to monitored conditions, a finding that may have implications in averting adverse reactions.

Interestingly, the only exception was when using Q1-L to the calf without monitoring. In this case the mean force was only 0.02 N higher than with monitoring; however, the SD was substantially greater and the range broader with an absolute difference between high and low of 10.0 N without monitoring, compared to 2.2 N with monitoring. Since the absolute mean difference approached zero in this case, the effect size was diminished. This instance illustrates a limitation of relying solely on mean differences, which do not capture distributional characteristics. Measures such as SD, range, and visual tools like scatter plots are essential to fully represent consistency and variability in clinical research.

It is necessary to consider variability in force delivery from the perspective of the individual’s experience. For example, as illustrated on the Bland-Altman plots, when using Q2-D to the calf without monitoring, one participant received a “medium” average peak force of 39.9 N while another received 9.0 N [30.9 N spread], but with monitoring the range narrowed from 21.2 N high to 16.0 N low [5.2 N spread]. In another example, from the perspective of a single individual’s experience, one clinician applied a self-perceived level of force of 46.48 N when using Q2-D without monitoring on the back, while another applied 16.33 N, representing an absolute difference of 30.2 N; but monitoring narrowed the spread from a 20.33 N high to 19.19 N low, a smaller absolute difference of 1.1 N. The interplay between subject perspective/preference and clinician perception of force applied on IASTM dosing warrants further investigation.

Unexpectedly, an inverse relationship between force and stroke frequency existed depending on the monitoring condition. Force was consistently applied at a lower magnitude but faster rate with monitoring, whereas without monitoring higher force was applied at a slower rate. Additionally, stroke frequency became more consistent during monitoring, indicating that monitoring force alone may exert a stabilizing effect. This may reflect potential force–stroke frequency interactions and influences on motor learning and control which merit deeper examination [46].

Monitoring had less effect on the consistency of IASTM application angles. Angles may reflect a clinician’s personal style, like how holding a pencil differently affects writing style. Clinicians’ hand grip (i.e., pencil vs. palm), device tilt, and stroking methods may influence angles. Observations revealed some clinicians held Q1-L tilted more to the side compared to vertically or inclined Q2-D more forward of the perpendicular (normal) to the skin. Also, some clinicians had bi-directional stroking methods, like swiping butter on bread, using back and forth motions with the device tip/blade versus a unidirectional motion pattern which altered angles. In-depth motion analysis of IASTM stroke application methods and patterns is implicated.

Device type influences the IASTM force-motion profiles regardless of the monitoring status. Clinicians applied higher levels of force when using Q2-D than Q1-L in both regions, at a slower rate and steeper angle. Higher force application was expected with Q2-D due to the broader contact area of its blade, but not the impact on rate or angle. Q2-D showed greater variability (larger SD and wider range) across both regions with stronger impact from monitoring on reliability than with Q1-L. Therefore, the physical characteristics of IASTM devices (e.g., size, weight, beveling) should be considered in research and practice [8].

Regional differences were found with monitoring. Monitoring had a stabilizing effect on force and stroke frequency in both regions. Mostly higher force was delivered to the back than calf. Region appeared to have less impact on stroke frequency. Angle varied regionally regardless of monitoring status. Q1-L applied to the calf showed less differences between monitoring conditions, whereas the same device on the back exhibited significantly greater variability. Variations may reflect differences in regional contour, tissue composition, and associated densities and are important to consider across all body regions [47, 48].

Monitoring improved IASTM force consistency for both novice and experienced clinicians but a significant difference based on experience level was not found. Further research is needed to clarify the role of visual feedback in motor learning and control for manual therapy applications.

The minimal clinically important difference (MCID) for variations in IASTM force remains undetermined, with existing literature emphasizing methodological heterogeneity and the absence of standardized dosing parameters. Regional differences in tissue sensitivity likely contribute to this uncertainty, as mechanoreceptor innervation density and receptive-field size vary across body sites, making sensation highly complex. Sensitivity to mechanical stimulus is multi-factorial, affected by age, sex, posture, modality, skin type (glabrous vs. hairy), disease, and stimulus type/direction, which complicates MCID determination [48, 49]. By way of analogy, 1 *N* = 102 g ≈ 0.25 pounds (1/4 lbs.). In practical context, healthy people can detect forces as low as 10 g (0.1 N) on the bottom of the foot and 8.5 g (0.08 N) on the hand [50, 51], and can on average discriminate a 5% increase or decrease in force applied to highly sensitive areas, whereas the back approaches a 10% differential [52]. Further consideration of the MCID for IASTM force–motion parameters are needed.

Results of this study do not imply optimal dosing for treatment efficacy or effectiveness. The relationship between consistency and clinical outcomes remains unclear and optimal dosing parameters are unknown. Exploration should be expanded to determine generalizability of findings to other body regions and patient populations. Nonetheless, reliable IASTM force application is fundamental to precision-based manual therapy.

Study findings showing real-time monitoring can reduce IASTM applied force variations have meaningful implications for education, research, and practice fidelity, helping to establish a reliable foundation for developing protocols that support evidence-based care. Reliable IASTM metrics are especially critical when patient care is shared among clinicians and for assessing changes in applied force between sessions.

This study has several limitations. Reliability testing was conducted only on younger participants within a restricted BMI range and no injury or pain, which may limit the generalizability of findings to broader populations. Also, testing was limited to two body regions, which may not translate to reliability in other areas since force application may be influenced by site, structure depth, and tissue type. Only female clinicians participated to reduce variability; however, potential gender differences could exist in force application. Additionally, testing was limited to linear IASTM stroke patterns, such as those used in cross-fiber massage or strumming, whereas curved patterns (e.g., fanning and sweeping), commonly employed in clinical practice, may introduce greater variability and warrant investigation. Since testing was limited to a single type of IASTM, findings may not extend to other tool sets with different edge designs and handle configurations.

Furthermore, targeted IASTM force levels used in this study may not be generalizable to clinical conditions. The intent of using targeted force is not to advocate standardized protocols as an ideal for care, but to support consistency in force application as individualized to the patient. Clinicians were allowed to apply force using their preferred stroke frequency, angle, and grip, with the goal of preserving their naturalistic practice style; however, controlling these parameters during research may further reduce variability and improve reproducibility.

Future research should investigate the influence of monitoring IASTM force-motion profiles on reliability across diverse musculoskeletal conditions, age groups, and body compositions, as applied by both male and female clinicians. To date, no studies have correlated the reliability of manual therapy applications, including IASTM, with clinical outcomes. In addition, the effects of real-time monitoring on potential constraints of clinician movement patterns, ergonomics, and potential overuse risk remain unknown, warranting investigation. Future studies may also examine how the frequency of visual monitoring influences force accuracy in relation to clinician attention to patient response. Prior work on AI-enabled deep learning model has expanded IASTM-motion performance classification [53], showing 93.2% (*n* = 5) accuracy in recognizing recorded curved vs. linear IASTM stroke motion patterns, opening an avenue for expansion and exploration.

Enhanced reliability and fidelity in IASTM application can enhance protocol development and support investigations exploring dose response on biological and functional outcomes. Progress requires force-quantifying instrumentation in clinical trials, standardized reporting of dose parameters, and outcome-linked analyses to determine perceptible and meaningful thresholds.

In summary, this study explored intra- and inter-examiner reliability of IASTM 3D force–motion parameters in humans. Findings support manual therapy practice by demonstrating that real-time visual monitoring significantly enhances consistency in IASTM 3D force application across devices, body regions, and sessions, regardless of experience level. Future research should determine whether enhanced reliability translates to better treatment efficacy.

## Conclusion

Objective IASTM force–motion parameters support evidence‑based manual therapy and may enhance the consistency of clinical application. In this study, real‑time visual monitoring of 3D force levels improved within‑clinician consistency and between‑clinician agreement across devices, sessions, and body regions. Integrating quantitative IASTM metrics with visual feedback can support individualized, precision rehabilitation while complementing patient input and clinician judgment. These findings have implications for personalized clinical practice, training, and research.

## Data Availability

The datasets used and/or analyzed during the current study are available from the corresponding author on reasonable request.

## Abbreviations

3D: Three dimensional, Tri-Axial
BMI: Body mass index
DPT: Doctor of physical therapy
FBM: Force based manipulation
GVI: Graphic visual interface
IASTM: Instrument assisted soft tissue manipulation
MCID: Minimal clinically important difference
MSK: Musculoskeletal
NIH: National institute of health
Q1-L: Q1-L localizing device applicator
Q2-D: Q2-D dispersive device applicator
QSTM^®^: Quantifiable soft tissue manipulation ^®^
SD(s): Standard deviation(s)
STM: Soft tissue manipulation
W: With
WO: Without
